# Mutation Spectrum Comparison between Benign Breast Lesion Cohort, Unselected Cancer Cohort and High-Risk Breast Cancer Cohort

**DOI:** 10.3390/cancers16173066

**Published:** 2024-09-03

**Authors:** Ava Kwong, Cecilia Y. S. Ho, Henry C. M. Leung, Amy W. S. Leung, Chun-Hang Au, Edmond S. K. Ma

**Affiliations:** 1 Division of Breast Surgery, Department of Surgery, The University of Hong Kong, Hong Kong SAR, China; 2 Hong Kong Hereditary Breast Cancer Family Registry, Hong Kong SAR, China; 3 Cancer Genetics Centre, Breast Surgery Centre, Surgery Centre, Hong Kong Sanatorium & Hospital, Hong Kong SAR, China; 4 Department of Pathology, Division of Molecular Pathology, Hong Kong Sanatorium & Hospital, Hong Kong SAR, China

**Keywords:** *BRCA*, hereditary breast cancers, Chinese, germline mutation

## Abstract

**Simple Summary:**

Germline mutation rates and spectra across three cohorts of Chinese patients: a high-risk breast cancer cohort, an unselected breast cancer cohort, and a benign breast lesion cohort were compared. The high-risk cohort had the highest mutation rate at 11.9%, while the unselected cancer and benign lesion cohorts had rates of 6.5% and 8.1%, respectively. Notably, 29.3% of the unselected breast cancer patients met genetic testing criteria, and this subgroup had a mutation rate similar to the high-risk cohort. High-penetrance gene mutations were only found in the high-risk and unselected cancer cohorts. Unexpectedly, a 2% mutation rate was observed in the benign lesion cohort. These findings highlight the need for broader genetic testing of all breast cancer patients, not just those deemed high-risk, to identify more individuals harboring clinically relevant germline mutations.

**Abstract:**

Mutation study for high-risk breast and ovarian cancer (HBOC) has been extensively studied in patients of different ethnicities. Here we compared the germline mutation rate and mutation spectrum of patients (*n* = 4341) with benign breast diseases or breast cancers, with and without other risk factors. Three cohorts of Chinese patients were recruited. The first cohort, high-risk cohort (HR, *n* = 3935) included high-risk breast cancer patients fulfilling high-risk HBOC criteria and who are recruited at our genetics clinic. The second cohort, unselected cancer cohort (CC, *n* = 307) was from general recruitment of patients with breast cancer at breast surgery clinics. The third cohort, benign breast lesion cohort (NC, *n* = 99) comprised 99 patients with benign breast diseases such as fibroadenoma, fibroadenomatoid hyperplasia, and intraductal papilloma. Thirty HBOC related genes were sequenced on the above-mentioned patient cohorts. The germline mutation rates of HR, CC, and NC cohort were 11.9%, 6.5%, and 8.1%, respectively. In the CC cohort, 29.3% (90/307) of patients fulfilled the National Comprehensive Cancer Network (NCCN) high-risk genetic test criteria 2022 v.2. The mutation rate for this group of patients was 11.1%, similar to that of the HR cohort, while the mutation rate for those not fulfilling testing criteria was 4.6%, like that of the NC cohort. High penetrance genes (*BRCA1/2*, *CDH1*, *PALB2*, *PTEN,* and *TP53*) mutations were only found in the HR (10.6%) and CC (3.3%) cohorts but were not found in the NC cohort. *ATM*, *BRIP1*, *RAD51C,* and *RAD51D* mutations were identified in all cohorts. *RAD51C* and *RAD51D* mutations showed conflicting penetrance. An unexpectedly high mutation rate of total 2% was found in the NC cohort but it was only 0.3% and 0.5% in the HR cohort and CC cohort, respectively. Our results show a clinical need to enhance genetic testing of unselected breast cancer patients to identify the high-risk patients.

## 1. Introduction

PARP inhibitors, olaparib and talazoparib, have been approved for the treatment of metastatic breast cancer in germline *BRCA1/2* mutation (gBRCAm) carriers [1,2]. The recent OlympiA trial showed improved progression-free survival (PFS) and distant disease-free survival with adjuvant olaparib for gBRCAm carriers with HER2-negative high-risk early-stage breast cancer [3]. The use of PARP inhibitor resulted in a significant improvement in PFS and health-related quality of life in gBRCAm carriers compared to non-platinum based single-agent chemotherapy [4,5]. Germline mutations in homologous recombination repair (HRR) genes were also listed as an inclusion criterion for the trials evaluating the effectiveness of talazoparib in HER2-negative breast cancer [6]. Advances in molecular genetics have identified pathogenic or likely pathogenic (P/LP) variants in several high to moderate-penetrance genes involved in regulating cell growth and/or DNA repair [7,8,9] that are associated with inherited susceptibility to breast, ovarian, prostate, colon, and pancreatic cancers (e.g., *BRCA1/2*, *TP53*, *CDH1*, *PTEN,* and *PALB2*), characterized by an early disease onset and exhibiting an autosomal dominant inheritance pattern [10,11,12]. With the technological development in next-generation sequencing (NGS), the availability of NGS has been extended to many developing countries and the cost of testing has been substantially reduced [12]. Multigene panel testing has become a common option to detect disease associated genetic mutation in hereditary breast cancer patients and their relatives. In 2014, King and her co-workers were the first group to advocate for population-based germline *BRCA1/2* screening for all women [13], but they met with controversy. In 2019, the American Society of Breast Surgeons published a consensus statement on genetic testing for all patients with a personal history of breast cancer [14]. The American College of Medical Genetics and Genomics (ACMG) also suggested subsequently evaluating the need for germline genetic testing on all patients with breast cancer [15]. In 2020, a Mayo Clinic study proposed a fusion approach for genetic testing in breast cancer patients. The study recommended testing all women diagnosed before the age of 65 and following NCCN testing criteria for those diagnosed after 65 [16]. However, the NCCN guideline for germline genetic testing has favored a more restricted approach, recommending testing only for high-risk patients due to the low rate of positive detections [17]. It was not until 2021, in guideline 2021 v.1, that the criteria were loosened by NCCN to include more breast cancer patients for genetic screenings to aid in PARP inhibitor treatment decisions [17]. In the 2023 v.1 update, NCCN further changed their testing criteria, expanding them to include even more breast cancer patients for genetic screenings based on diagnosis age and family histories [18]. A more recent update by ASCO Society of Surgical Oncology also recommend testing all newly diagnosed patients with breast cancer ≤65 years (with stage I–III or de novo stage IV/metastatic disease), and the testing criteria only apply for patients >65 years based on personal cancer history, family history, and ancestry [19].

Here, we conducted a local examination of germline mutations in consecutive patients recruited from three different specialist centers (high-risk genetic clinic, breast surgery center, and general breast clinic) to compare mutation detection rates and spectra in Chinese populations. Our results show a clinical need to enhance genetic testing for all patients with breast cancer to identify high-risk mutation carriers.

## 2. Methods

### 2.1. Participants and Selection Criteria

Germline mutation screening was performed on 4341 Chinese individuals recruited through the Hong Kong Hereditary Breast Cancer Family Registry from March 2007 to August 2022. These individuals were from three cohorts of Chinese patients. The first cohort, the HBOC high-risk cohort (HR, *n* = 3935), comprised high-risk breast cancer patients who fulfilled the testing criteria described in our previous paper [20], including patients meeting any of the following criteria: (1) they had been diagnosed with breast cancer at any age and had at least one first- or second-degree relative with breast and/or ovarian cancer, regardless of age; (2) they had been diagnosed with breast cancer at or before 45 years of age; (3) they had bilateral breast cancer; (4) they had triple-negative breast cancer; or (5) they were male with breast cancer. The second cohort, the unselected cancer cohort (CC, *n* = 307), consisted of patients with breast cancer who were recruited from general breast surgery clinics, regardless of any selection criteria. The third cohort, the benign breast lesion cohort (NC, *n* = 99), included 99 patients with benign breast diseases or abnormalities such as fibroadenoma, fibroadenomatoid hyperplasia, and intraductal papilloma. All participants recruited gave written informed consent for the study. The research was conducted in accordance with the Declaration of Helsinki.

### 2.2. DNA Extraction and Sequencing

Genomic DNA extraction from the peripheral blood was performed by the QIAamp DNA Blood Mini Kit (Qiagen, Hilden, Germany) or the QIAsymphony DNA Mini Kit (Qiagen, Hilden, Germany) according to the manufacturer’s instructions. Genomic DNA were sequenced by Color Genomics with a 30 HBOC gene panel or in-house with 93-genes DHS-001Z human breast cancer panel by the Qiagen breast cancer panel (Qiagen, Hilden, Germany) on MiSeq/NextSeq/NovaSeq (Illumina, San Diego, CA, USA) instruments, and the sequencing results of 30 HBOC genes were analyzed. The minimum sequencing depth was 50× and median coverage was 200–300×. All detected pathogenic variants were further validated by conventional Sanger bi-directional DNA sequencing.

### 2.3. Variant Interpretation and Annotation

Variants calling bioinformatics was performed as previously described [20,21]. Paired sequencing reads were mapped to human reference genome sequence GRCh37/hg19. Variants with a minor allele frequency of at least 1% reported by the 1000 Genomes Project [22] were excluded from manual variant curation. Variants were described according to the recommendations of the Human Genome Variation Society (HGVS) nomenclature (http://www.HGVS.org/varnomen). The variant descriptions were further cross-checked with Mutalyzer Name Checker (http://mutalyzer.nl).

### 2.4. Statistical Analysis

Multiple comparisons were conducted among cohorts. One-way analysis of variance (ANOVA) followed by Fisher’s least significant difference (LSD) were applied to compare continuous variables. Fisher’s exact test was used to study the independence of categorical variables between cohorts. The limit of significance for all analyses was defined as a *p*-value of <0.05. Data analyses were performed using the statistical software R (version 3.4.2) [23].

## 3. Results

### 3.1. Patient Characteristics of the Cohorts

We recruited 3935 HBOC high-risk (HR) patients (*n* = 3935) from our genetic clinic. These patients fulfilled the NCCN 2022.v2 testing criteria during their first referral, with a median age of diagnosis of 47.6 years, ranging from 19.3 to 95.8. Over 70% of the breast cancers from these patients were hormonal positive invasive ductal carcinoma. Of these patients, 40.8% had a family history of breast cancer, and 30.7% had a family history of *BRCA*-related cancers.

Our unselected breast cancer control cohort (CC, *n* = 307) consisted of patients randomly recruited from our breast surgery cancer center. Among these patients, 29.3% (90/307) fulfilled the NCCN 2022.v2 testing guidelines, while 70.7% (217/307) did not meet any high-risk criteria. The median age of diagnosis for those fulfilling the testing criteria was 55.3 years, ranging from 32.1 to 90.6, while those not fulfilling the criteria had an older median age of diagnosis at 59.8 years, ranging from 45.0 to 82.5. The histology of both groups was predominantly invasive ductal carcinoma, at 90% and 82.5%, respectively. The major differences between those who did or did not fulfill the NCCN 2022.v2 testing guideline were in TNBC subtype and family history of breast or ovarian cancers (Table 1). In the CC cohort who fulfilled the NCCN criteria, 22.5% of the patients had TNBC, 36.7% had a family history of breast cancer, and 5.6% had a family history of ovarian cancer.

Of the 99 benign breast patients in the NC cohort, the median age of diagnosis of breast benign disease was 47.0 years, ranging from 20.8 to 84.9. Most of the breast diseases in these patients were fibro-epithelial tumors (55.6%), such as fibroadenoma, phyllodes tumor, and fibroadenomatoid change. Patients numbering 14.1% had intraductal papilloma, ductal adenoma, or fibromatosis, and were classified as other benign tumors. Patients numbering 11.1% had fibrocystic changes in their breasts, and 8.1% had other non-neoplastic benign breast problems. Only 6.1% and 1.1% of these NC patients had family history of breast cancer or ovarian cancer, respectively. Clinicopathological characteristics of the patient cohorts are listed in Table 1.

### 3.2. Germline Mutations between Cohorts

**Figure 1:**
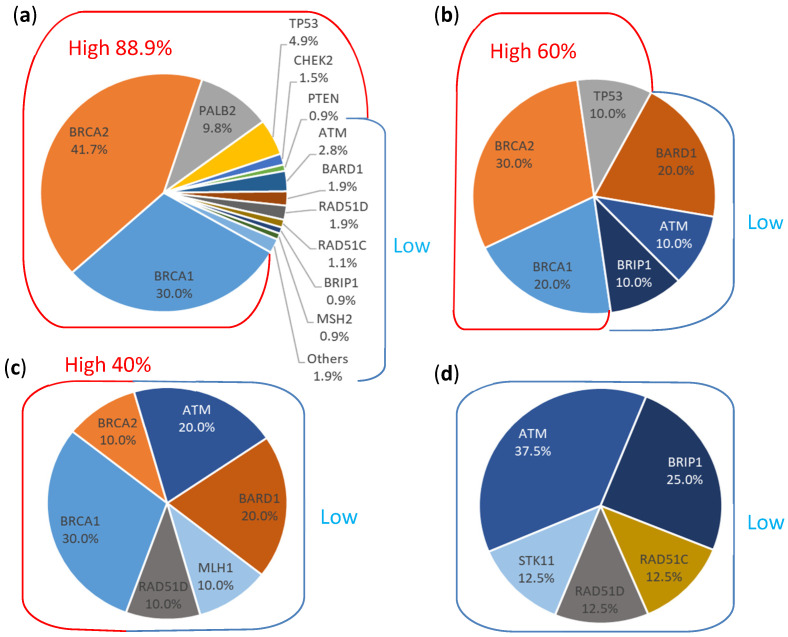
Distributions of pathogenic or likely pathogenic mutations identified in different cohorts. (**a**) High-risk cohort (HR, *n* = 3935) comprised high-risk breast cancer patients fulfilling the NCCN genetic testing criteria. (**b**) Unselected breast cancer cohort (*n* = 90) who fulfilled the NCCN genetic testing criteria. (**c**) Unselected breast cancer cohort (*n* = 217) who did not fulfill the NCCN genetic testing criteria. (**d**) Benign breast lesion cohort (NC, *n* = 99) comprised of patients with benign breast diseases. Red line: high penetrance genes; blue line: moderate to low penetrance genes.

The germline mutation rates of having pathogenic or likely pathogenic mutation variant among 30 genes of the HR, CC, and NC cohort were 11.9%, 6.5%, and 8.1%, respectively. In the CC cohort, the mutation rate for patients who fulfilled the NCCN genetic test criteria was 11.1%, similar to that of the HR cohort, while the mutation rate for those who did not fulfill the testing criteria was 4.6%, like that of the NC cohort. High penetrance gene mutations (*BRCA1/2*, *CDH1*, *PALB2*, *PTEN,* and *TP53*) were found in 10.6% of HR patients and in 3.3% of the CC cohort (with mutation rates of 6.7% and 1.8% for those who did and did not fulfill the NCCN criteria, respectively), but none were found in the NC cohort (Table 2). The most frequently mutated gene was *BRCA2* in the HR and CC (met criteria) cohorts, with the mutation rates of 5% and 3.3%, respectively. *BRCA2* mutations were also identified in the CC non-high-risk cohort but the mutation rate was only 0.5%, and no *BRCA2* mutation was found in the NC cohort. In other moderate and low penetrance genes, mutations were found in only 1.3% of HR patients and in 3.3% of the CC cohort (with mutation rates of 4.4% and 2.8% for those who did and did not fulfill the NCCN criteria, respectively), but in 8.1% of the NC cohort (Table 2). Mutations in *ATM*, *BRIP1*, *RAD51C,* and *RAD51D* were identified in all cohorts. *RAD51C* and *RAD51D* mutations showed conflicting penetrance. An unexpectedly high mutation rate of 2% was found in the NC cohort, but it was only 0.3% and 0.5% in the HR cohort and CC non-high-risk cohorts, respectively (see Table 2 and Figure 1). Detailed mutation spectra from each cohort are listed in Supplementary Table S1.

## 4. Discussion

NGS multiple-gene panel testing has improved the detection rates of mutations and provided a cost-effective cancer risk assessment. The identification of germline pathogenic or likely pathogenic mutations in cancer susceptibility genes has considerable significant implications for cancer prevention, early detection, treatment (such as with poly ADP ribose polymerase inhibitors, PARPi), and management, both for patients and for their relatives. Despite the benefits of molecular screening, many breast cancer patients never undergo testing due to the current adopted testing criteria, such as England NICE test criteria, which predominantly rely on family history and pathology of the tumors. In a study of 35,000 patients from multiple ancestries with unselected breast cancer, a mutation rate of 9.3% was found using a 25-gene panel, and the positive rate ranged from 7.2% to 11.5% based on ancestry [24]. In another study of a consecutive series of 10,000 cancer patients and unaffected individuals undergoing 29-genes NGS testing, with 82% of patients being Caucasian, 9.0% of patients were found to carry at least one pathogenic or likely pathogenic variant, with 51.2% of these mutations being in high penetrance genes [25]. In another multicenter study involving 2984 patients with unselected personal cancer history and family history who underwent an 80-genes panel test, P/LP variant was found in 13.3% of patients, with 5% of the mutations being identified from highly penetrant genes, and more than half of the identified variants from genes with moderate or low penetrance [26]. In Asian populations, studies were done on unselected breast cancer germline mutation spectra, the mutation rates ranged from 1.5% to 2.7% for *BRCA1* and 2.4% to 3.8% for *BRCA2* [27]. For Asian HBOC patients, mutation rates ranged from 1.3% to 14.6% for *BRCA1* and 3.2% to 10.8% for *BRCA2* [27]. In another study of 13,129 cancer-free Chinese individuals, the *BRCA1* and *BRCA2* mutation rates were 0.2% and 0.4%, respectively [28]. At least 10% of unselected breast cancer patients in China carry pathogenic variants in cancer susceptibility genes [29]. These findings demonstrate that at least 1.5% to 3.8% of *BRCA1/2* mutations were found in breast cancer patients without criteria selection. In our study, we clearly showed a 1.8% mutation rate of high penetrance genes, with 1.4% from *BRCA1* and 0.5% from *BRCA2* (Table 2), concealed in non-high-risk breast cancer patients, who needed alternations in management during their ongoing healthcare.

Among the mutation spectra in moderate and low penetrance genes, *ATM*, *BRIP1*, *RAD51C,* and *RAD51D* mutations were identified in all cohorts. A systemic review showed that the prevalence of the deletion, insertion, substitution mutation variants in *ATM* are associated with breast cancer [30]. In United States, a high prevalence of 6.6% was found in *ATM* [31], and many *ATM* mutations have been described and associated with a moderate risk of developing breast cancer [32,33,34,35]. In Asian populations, the pathogenic mutation frequency of *ATM* was around 1% for HBOC patients and similar frequencies were seen in unselected breast cancer patients. However, the mutation percentage increased to 3% for those with benign breast disease (Table 3).

Germline mutations were also commonly found in patients with breast fibroadenomas. An *ATM* c.8246A > T; p. (Lys2749Ile) germline mutation carrier was identified in one out of 12 fibroadenoma patients in the Chinese population [36]. Most studies from Sweden, Finland, and Denmark reported no association between common variants in the *ATM* gene and breast cancer susceptibility [30]. This observation was also seen in our Chinese population.

*RAD51C* and *RAD51D* mutations showed conflicting penetrance. An unexpectedly high mutation rate of 2.0% was found in the NC cohort, but it was only 0.36% and 0.33% in the HR and CC cohorts, respectively. Most of our *RAD51D* mutation carriers carried c.270_271dupTA; p. (Lys91Ilefs*13), which is a well-known mutation, especially in the Asian population [37,38]. In the Genome Aggregation Database (gnomAD), this variant was observed in 14/18, 394 (0.076%) individuals in the East Asian population but not in other populations. Management of *RAD51C* and *RAD51D* mutation carriers has been controversial. In ACMG published guidance for reporting secondary findings in the context of clinical exome and genome sequencing, *RAD51C* and *RAD51D* mutations were not listed in SF v3.1 and were no longer being reported as secondary findings, with comments on moderate risk of primarily later-onset breast cancer and low penetrance for ovarian cancer [39]. In the NCCN management guidelines 2023 v.3 [17], the absolute risk of breast cancer for *RAD51C* and *RAD51D* mutation carriers was revised form 15–40% to 20–40%, and management was also revised to include annual mammograms and consideration of breast MRI with contrast starting at age 40. For ovarian cancer, management on *RAD51C* and *RAD51D* mutation carriers was also revised, with patients now being recommended for risk-reducing salpingo-oophorectomy (RRSO) at age 45–50, rather than just being considered for it. The incomplete penetrance of *RAD51D* pathogenic variants has been demonstrated from a clinical, molecular pathology, and in vitro perspective (manuscript under review). Given the incomplete penetrance and high mutation rate observed in the NC cohort, a population screening approach is particularly important.

This study had some limitations. Multiple comparisons were made using one-way ANOVA with LSD tests between age distributions in the four cohorts, which showed a significant difference (*p* < 0.001). The HR cohort was found to be relatively younger than the CC (fulfilled NCCN) cohort, resulting in a higher mutation frequency in high penetrance genes, even though they had the same grouping criteria. Additionally, the family history of *BRCA*-related cancers in these two cohorts also showed a *p*-value of 0.0007. In the HR cohort, 30.7% of patients had family members with *BRCA*-related cancers other than breast and ovarian cancer (such as prostate cancer, pancreatic cancer, colorectal cancer, stomach cancer, melanoma, and cholangiocarcinoma), while only 14.4% of CC (fulfilled NCCN) patients had such family histories. This may suggest that these *BRCA*-related cancers are often missed by clinicians, and patients are not referred to high-risk genetic clinics for genetic testing. This reveals a problem in the referral system, as patients in the CC (fulfilled NCCN) cohort should have been referred for genetic testing. In one of our previous studies, we interviewed medical specialists in breast and ovarian cancer in Hong Kong and found that 8.5% of them may hesitate to refer patients for genetic test, and 9.9% of them do not have the related information and resources to refer patients to such testing [40].

**Table 3:** Mutation frequencies in different cohorts (HBOC, unselected breast cancer patient, benign breast disease patient, and normal control) from Asian countries on commonly tested HBOC related genes besides *BRCA1/2*.

	HBOC						Unselected Breast Cancer		Benign Breast	Normal Control
	Japan [41]	Taiwan [42]	China [43]	Singapore [44]	Korea [45]	Hong Kong	China [28]	Hong Kong	Hong Kong	China [28]
Panel	30 Genes	20 Genes	22 Genes	Mixed	35 Genes	Mixed	15 Genes	30 Genes	30 Genes	15 Genes
*N* =	568	480	481	460	120	3935	8067	307	101	13,129
	%	%	%	%	%	%	%	%	%	%
*BRCA1*	Neg	1.25	14.6	14.1	Neg	3.58	1.81	1.63	0	0.19
*BRCA2*	Neg	7.08	5	9.3	Neg	4.98	3.52	1.3	0	0.35
*PALB2*	1.23	1.88	1.7	0.4	2.5	1.2	0.71	0	0	0.14
*TP53*	0	0.21	0.6	1.3	1.7	0.6	0.38	0.33	0	0.02
*PTEN*	0	0	0.4	0	0	0.1	0.06	0	0	0
*CDH1*	0	0	0	0.22	0	0	0.01	0	0	0
*ATM*	0.88	0.63	1	0.4	0	0.3	0.38	0.98	3	0.18
*BARD1*	0.88	0.21	0	0	1.7	0.2	0.19	1.3	0	0.06
*RAD51D*	0.7	1.25	0.4	0.22	0	0.2	0.38	0.33	1	0.18
*BRIP1*	0.53	0.21	0.8	0.4	1.7	0.1	0.14	0.33	2	0.22
*RAD51C*	0.53	0.42	0	0.22	0	0.1	0.02	0	1	0.17
*CHEK2*	0.18	0	0	0.22	0	0.2	0.32	0	0	0.13
*NBN*	0	0	0.4	0.22	0	0	0.07	0	0	0.04
*STK11*	0	0	0	0	0	0	0.01	0	1	0.01
*RAD50*	0.18	0.21	0.4	0.22	0	NT	0.26	NT	NT	0.24
*PMS2*	0	0.21	0	0	0	0.03	NT	0	0	NT
*MSH2*	0	0	0.4	0.22	0	0.1	NT	0	0	NT
*MLH1*	0	0	0	0.7	0	0	NT	0.33	0	NT
*MSH6*	0	0	0	0	0	0.03	NT	0	0	NT
*MUTYH*	NT	NT	0.6	1.5	0	0.13	NT	0	0	NT
*BLM*	0.7	NT	NT	NT	0	NT	NT	NT	NT	NT

NT: Not tested.

Given that mutations from high penetrance genes were identified not only in the high-risk cohort but also in unselected breast cancer control, genetics screening on unselected breast cancer approach is particularly important, especially on identifying patients who are suitable for use of PARPi [4]. However, financial support is another concern of limitation for genetic tests; many counties often require a patient to meet stringent genetic testing criteria before reimbursement while the test is not supported by governments in many well-developed Asian countries including Hong Kong, Singapore, Malaysia, and Taiwan, while Korea and Japan only support patients suspected for HBOC. To balance between the limitations between strategic and universal screening, the Mayo Clinic suggested a hybrid approach of testing all women diagnosed with breast cancer before the age of 65 years and using NCCN criteria for older patients [16]. In 19 of our identified pathogenic mutation carriers from non-high-risk breast cancer patients, 10 of them carry mutation from high penetrance genes, 3 out of 10 were aged over 65, and among them two of them are *BCRA1* mutations carriers. From this finding, the hybrid approach of testing is not applicable in the Chinese population. Thus, we propose performing universal germline mutation screening on all patients with breast cancer as a favorable approach to implement in the Chinese population.

## 5. Conclusions

In summary, our study across these three clinical settings revealed the necessity to broaden genetic testing protocols for breast cancer patients in the Chinese population. Implementing more comprehensive testing strategies could lead to better detection of high-risk germline mutations and improve clinical management for these individuals.

## Figures and Tables

**Table 1:** Clinicopathological characteristics of patient cohorts.

	High-Risk (HR) Cancer Patient	Unselected Cancer Control (CC) Patient		Benign Disease (NC) Patient	*p*-Value
		Fulfill NCCN Testing Criteria	Not Fulfill NCCN Testing Criteria		
	*n* = 3935	*n* = 90	*n* = 217	*n* = 99	
**Age at recruitment (Median/Range)**					
Mean	49.4	55.7	60.9	45.9	-
Median	47.6	55.3	59.8	47.0	-
Range	19.3–95.8	32.1–90.6	45.0–82.5	20.8–84.9	-
**No. of patients identified P/LP (30 genes)**	463 ^	10	10 *	8	-
**Overall mutation %**	11.9% (469/3935)	6.5% (20/307)		8.1% (8/99)	0.0174
		11.1%	4.6%		
**Mutation % in high penetrance genes** (*BRCA1/2*, *PALB2*, *CDH1*, *PTEN*, *TP53*)	10.4% (410/3935)	6.7% (6/90)	1.8% (4/217)	0%	<0.0001
**Mutation % in moderate and low penetrance genes**	1.5% (59/3935)	4.4% (4/90)	2.8% (6/217)	8.1% (8/99)	<0.0001
**Personal breast disease**					
Breast cancer	3935	90	217	-	-
Fibro-epithelial tumors	-	-		-	55 (55.6%)	-
Other benign tumors					14 (14.1%)	
Fibrocystic changes					11 (11.1%)	
Other non-neoplastic					8 (8.1%)	
Inflammatory					6 (6.1%)	
Undefined					3 (3.0%)	
Congenital anomalies					2 (2.0%)	
**Histology**					
Ductal	3312 (70.4%)	81 (90%)	179 (82.5%)	-	-
In situ	771 (16.4%)	1 (1.1%)	2 (0.9%)	-	
Other	446 (9.5%)	8 (8. 9%)	35 (16.1%)	-	
Unclassified	174 (3.7%)	0 (0%)	1 (0.5%)	-	
**Molecular subtypes**					
Luminal A (Her2−)	2080 (52.9%)	51 (57.3%)	202 (94%)	-	-
Luminal B (Her2+)	476 (12.1%)	0 (0%)	9 (4.2%)	-	
Luminal A/B (Her2 unknown/equivocal)	221 (5.6%)	0 (0%)	1 (0.5%)	-	
TNBC	593 (15.1%)	20 (22.5%)	-	-	
**Histology (invasive) grade**					
Low	2078 (63.9%)	49 (57.0%)	151 (73.7%)	-	-
High	1174 (36.1%)	37 (43.0%)	54 (26.3%)	-	
No information	680	3	9	-	
**Stage of breast**					
0	824 (18.6%)	0 (0%)	0 (0%)	-	-
1	1643 (37.1%)	40 (45.5%)	83 (38.6%)	-	
2	1332 (30.0%)	31 (35.2%)	104 (48.4%)	-	
3	483 (10.9%)	17 (19.3%)	24 (11.2%)	-	
4	151 (3.4%)	0 (0%)	4 (1.9%)	-	
No information	270	2	2	-	
**Family history (1st or 2nd degree)**					
Breast cancer	1604 (40.8%)	33 (36.7%)	15 (6.9%)	6 (6.1%)	<0.0001
Ovarian cancer	195 (5.0%)	5 (5.6%)	0 (0.0%)	1 (1.1%)	0.0047
Other *BRCA* related cancer ^$^	1208 (30.7%)	13 (14.4%)	29 (13.4%)	19 (19.2%)	<0.0001

^ 7 probands & * 1 proband carried double pathogenic mutations. ^$^ Other *BRCA* related cancer: prostate cancer, pancreatic cancer, colorectal cancer, stomach cancer, melanoma, cholangiocarcinoma.

**Table 2:** Mutation distributions among cohorts.

	High-Risk Breast Cancer Patient (HR)	Unselected Cancer Cohort (CC)		Patient with Benign Breast Disease (NC)
		High-Risk Breast Cancer Patient (Met NCCN 2022 v2 Criteria)	Non High-Risk Breast Cancer Patient (Not Met NCCN 2022 v2 Criteria)	Not in NCCN 2022 v2 Criteria Based on FH
*n*	3935	90	217	99
Identified P/LP	463 ^	10	10 *	8
Overall mutation (%)	11.9% (469/3935)	6.5% (20/307)		8.1%
		11.1%	4.6%	
High penetrance (%)	10.6% (417/3935)	3.3% (10/307)		0%
		6.7% (6/90)	1.8% (4/217)	
*BRCA1*	3.6%	2.2%	1.4%	0%
*BRCA2*	5.0%	3.3%	0.5%	0%
*CDH1*	0%	0%	0%	0%
*PALB2*	1.2%	0%	0%	0%
*PTEN*	0.1%	0%	0%	0%
*TP53*	0.6%	1.1%	0%	0%
Moderate & low penetrance (%)	1.3% (52/3935)	3.3% (10/307)		8.1%
		4.4% (4/90)	2.8% (6/217)	
*RAD51C*	0.1%	0%	0%	1%
*RAD51D*	0.2%	0%	0.5%	1%

^ 7 probands & * 1 proband carried double pathogenic mutations.

## Data Availability

The dataset supporting the conclusions of this article is included within the article and its Supplementary Files.

## Supplementary Materials

The following supporting information can be downloaded at: https://www.mdpi.com/article/10.3390/cancers16173066/s1, Table S1: Mutation Spectrum from different cohort.
