# Supplementary material for: Mutation Spectrum Comparison between Benign Breast Lesion Cohort, Unselected Cancer Cohort and High-Risk Breast Cancer Cohort
Source: Cancers (Basel). 2024 Sep 3;16(17):3066. doi: 10.3390/cancers16173066 (PMC11393947; doi:10.3390/cancers16173066)
Supplement: Supplementary file 1 [file cancers-16-03066-s001.zip › cancers-3162067-SI.pdf]

Supplementary Table S1. Mutation Spectrum from different cohort.

Cohort	Genes penetrance	Gene mutation	Mutation Variant	Frequency
High Risk (HR) Cancer Patient	High^	*BRCA1*	c.-101196_4185+776del	1
			c.-39115_548-550del	1
			c.-3659_80+549del	1
			c.34C>T; p.Gln12*	1
			c.53T>C; p.Met18Thr	1
			c.66dupA; p.Glu23Argfs*18	1
			c.116G>A; p.Cys39Tyr	1
			c.121C>A; p.His41Asn	1
			c.134+1G>T; r.81_134del54; p.Cys27*	1
			c.134+5G>A; p.Cys27*	1
			c.135-2745_442-1597dup; r.135_441dup; p.Gln148Ilefs*20	1
			r.135_441dup; p.Gln148Ilefs*20	1
			r.442_547del; p.Gln148Aspfs*51	2
			c.183T>G; p.Cys61Trp	1
			c.190T>C; p.Cys64Arg	1
			c.213-12A>G; r.212_213insTTTAATTTCAG; p.Arg71Serfs*21	1
			c.212_212+1delGGinsAT; r.191_212del22; p.Cys64*	1
			c.212+1G>T; p.Cys64*	1
			c.212+3A>T; p.Cys64* & p.Phe46_Arg71del26	1
			c.220C>T; p.Gln74*	1
			c.280C>T; p.Gln94*	1
			c.431dupA; p.Asn144Lysfs*15	1
			r.442_670del229; p.Gln148Leufs*10	1
			c.470_471delCT; p.Ser157*	7
			c.502A>T; p.Lys168*	1
			c.505C>T; p.Gln169*	1
			c.547+428_4185+1319del; r.548_4185del; p.Gly183Alafs8*	1
			c.928C>T; p.Gln310*	1
			c.953delinsTGT; p.His318Leufs*24	1

			c.964delG; p.Ala322Leufs*19	2
			c.981_982delAT; p.Cys328*	5
			c.1058G>A; p.Trp353*	2
			c.1214C>G; p.Ser405*	1
			c.1465G>T; p.Glu489*	1
			c.1881_1884delCAGT; p.Ser628Glufs*3	2
			c.1898delC; p.Pro633Hisfs*18	1
			c.1961dupA; p.Tyr655Valfs*18	1
			c.2017delG; p.Glu673Asnfs*28	1
			c.2110_2111delAA; p.Asn704Cysfs*7	1
			c.2166delC; p.Asn723Ilefs*13	1
			c.2253_2254delGT; p.Met751Ilefs*10	1
			c.2577_2578insTT; p.Thr860Leufs*34	1
			c.2635G>T; p.Glu879*	4
			c.2695delG; p.Val899Serfs*101	1
			c.2764_2767delACAG; p.Thr922Leufs*77	1
			c.2866_2870delTCTCA; p.Ser956Valfs*13	1
			c.3214delC; p.Leu1072*	3
			c.3286C>T; p.Gln1096*	2
			c.3333delA; p.Glu1112Asnfs*5	2
			c.3342_3345delAGAA; p.Glu1115*	5
			c.3531dupT; p.Ser1178*	1
			c.3607C>T; p.Arg1203*	1
			c.3627dupA; p.Glu1210Argfs*9	1
			c.3648dupA; p.Ser1217Ilefs*2	1
			c.3756_3759delGTCT; p.Ser1253Argfs*10	2
			c.3767_3768delCA; p.Thr1256Argfs*10	1
			c.3858_3861delTGAG; p.Ser1286Argfs*20	2
			c.3869_3870delAA; p.Lys1290Metfs*4	1
			c.3893C>A; p.Ser1298*	1
			c.3916_3917delTT; p.Leu1306Aspfs*23	1
			c.3968_3971delAAAT; p.Gln1323Argfs*12	1
			c.4049dupG; p.Glu1352Glyfs*4	1
			c.4065_4068delTCAA; p.Asn1355Lysfs*10	2
			c.4148C>G; p.Ser1383*	1
			c.4372C>T; p.Gln1458*	5
			c.4656C>G; p.Tyr1552*	1

			c.4676-2A>G; r.4676_4706del31; p.Glu1559Alafs*32	1
			c.4695dupA; p.Ser1566Ilefs*8	1
			c.4780_4793del14; p.Pro1594Cysfs*23	1
			c.4903G>T; p.Glu1635*	1
			c.4986+5G>A; r.4986+1_4986+65ins; p.Met1663Valfs*14	1
			c.4986+891_*13331del	1
			c.4987-443_5278-2407del; r.4987_5277del291; p.Met1663_Lys1759del97	1
			c.5072C>A; p.Thr1691Lys	3
			c.5074+3A>G; r.4987_5074del; p.Val1665Serfs*8	1
			c.5075-1G>C; r.5075_5152del78; p.Asp1692_Trp1718delinsGly	1
			c.5089T>C; p.Cys1697Arg	7
			c.5091T>A; p.Cys1697*	1
			c.5155delG; p.Val1719*	1
			c.5165C>T; p.Ser1722Phe	1
			c.5193+1G>A; r.5153_5193del41; p.Trp1718Serfs*2	1
			c.5193+1G>C; p.Trp1718Serfs*2	1
			c.5193+716_5407-519del; r.5194_5406del; p.His1732_Thr1802del	1
			c.5194-671_5277+408del; r.5194_5277del; p.His1732_Lys1759del	1
			c.5202delT; p.Phe1734Leufs*31	1
			c.5266C>T; p.Gln1756*	1
			c.5282T>C; p.Phe1761Ser	1
			c.5335delC; p.Gln1779Asnfs*14	1
			c.5353C>T; p.Gln1785*	1
			c.5406+1_5406+3delGTA; p.Asp1778Glyfs*27	4
			c.5406+7A>G; r.5333_5406del74; p.Asp1778Glyfs*27	1
			c.5470_5477del; p.Ile1824Aspfs*3	1
			c.5503C>T; p.Arg1835*	1

			c.5511G>C; p.Trp1837Cys	1
			c.5521A>C; p.Ser1841Arg	3
			Del in exon 23-24	1
		BRCA2	c.-39-1_-39delGA	1
			c.262_263delCT; p.Leu88Alafs*12	1
			c.470_474delAGTCA; p.Lys157Serfs*24	4
			c.476-1G>A; r.426_516del91; p.Ser142Argfs*13	1
			c.476-3C>A; r.426_516del91; p.Ser142Argfs*13	1
			c.515A>G; r.476_516del; p.Val159Glyfs*10	1
			c.632-6T>G; r.632_681del50; p.Val211Glufs*10	1
			c.658_659delGT; p.Val220Ilefs*4	1
			c.682-1G>C; r.682_793del112; p.Asn228Aspfs*12	1
			c.722delA; p.Lys241Argfs*10	1
			c.724A>T; p.Lys242*	1
			c.755delA; p.Asp252Alafs*25	1
			c.771_775delTCAAA; p.Asn257Lysfs*17	1
			c.904dupA; p.Thr302Asnfs*3	1
			c.956delA; p.Asn319Ilefs*5	1
			c.994delA; p.Ile332Phefs*17	1
			c.1053delA; p.Lys351Asnfs*16	1
			c.1244delA; p.His415Leufs*15	1
			c.1261C>T; p.Gln421*	1
			c.1296_1297delGA; p.Asn433Glnfs*18	1
			c.1376T>G; p.Leu459*	1
			c.1454delinsTGTATT; p.Lys485Metfs*26	1
			c.1688G>A; p.Trp563*	1
			c.1813delA; p.Ile605Tyrfs*9	1
			c.1855C>T; p.Gln619*	1
			c.1888dupA; p.Thr630Asnfs*6	2
			c.2004delG; p.Lys669Asnfs*13	1
			c.2339C>G; p.Ser780*	1
			c.2442delC; p.Met815Trpfs*10	2
			c.2595delA; p.Glu866Lysfs*8	3

			c.2808_2811delACAA; p.Ala938Profs*21	15
			c.2870delA; p.Asn957Ilefs*3	2
			c.3109C>T; p.Gln1037*	33
			c.3202delG; p.Val1068Tyrfs*9	1
			c.3265C>T; p.Gln1089*	1
			c.3760G>T; p.Glu1254*	1
			c.3779dupT; p.Leu1260Phefs*5	1
			c.3836delA; p.Asn1279Metfs*5	1
			c.4121delA; p.Lys1374Argfs*14	1
			c.4169delT; p.Leu1390Trpfs*20	1
			c.4440T>G; p.Tyr1480*	2
			c.4563_4564delGT; p.Leu1522Glyfs*6	1
			c.4651C>T; p.Gln1551*	1
			c.5164_5165delAG; p.Ser1722Tyrfs*4	7
			c.5238dupT; p.Asn1747*	1
			c.5576_5579delTTAA; p.Ile1859Lysfs*3	1
			c.5578A>T; p.Lys1860*	2
			c.5644_5647delTCAA; p.Ser1882Lysfs*26	1
			c.5722_5723delCT; p.Leu1908Argfs*2	4
			c.5844_5856delTGATGTTAGTTTG; p.Cys1948Trpfs*11	1
			c.5851_5854delAGTT; p.Ser1951Trpfs*11	2
			c.5951_5952delAA; p.Lys1984Ilefs*18	1
			c.6040delG; p.Val2014Tyrfs*26	1
			c.6096dupT; p.Ile2033Tyrfs*16	2
			c.6240dupA; p.Glu2081Argfs*4	1
			c.6443_6444delCT; p.Ser2148Tyrfs*2	1
			c.6486_6489delACAA; p.Lys2162Asnfs*5	2
			c.6582delT; p.Ile2194Metfs*12	1
			c.6656C>G; p.Ser2219*	1
			c.6678delA; p.Ala2227Glnfs*2	1
			c.6698delC; p.Ala2233Valfs*8	1
			c.6990dupT; p.Thr2331Tyrfs*9	1
			c.6997_6998delGT; p.Val2333Thrfs*6	1
			c.7007G>A; r.6938_7007del70; p.Gly2313Alafs*31	5
			r.7008_8331del1324; p.Thr2337Phefs*43	1

			c.7133C>G; p.Ser2378*	2
			c.7409dupT; p.Thr2471Hisfs*4	1
			c.7435+508_7805+462del; p.Asp2479Glyfs*46	3
			c.7467dupT; p.Ile2490Tyrfs*7	1
			c.7471C>T; p.Gln2491*	1
			c.7490dupA; p.Lys2498Glufs*41	1
			c.7558C>T; p.Arg2520*	1
			c.7726G>T; p.Gly2576*	1
			c.7806-9T>G; p.Arg2602Serfs*49	1
			c.7816dupG; p.Asp2606Glyfs*12	1
			c.7878G>A; p.Trp2626*	7
			c.7976+1G>A; r.7806_7976del171; p.Ala2603_Arg2659del57	1
			c.7976+5G>A; r.7806_7976del171; p.Ala2603_Arg2659del57	1
			c.8009C>T; p.Ser2670Leu	2
			c.8023A>G; p.Met2676_Ile2778del	1
			c.8068_8069delGT; p.Val2690Phefs*2	2
			c.8162T>A; p.Leu2721His	1
			c.8208_8209insAG; p.Leu2737Serfs*2	1
			c.8245C>T; p.Gln2749*	1
			c.8331+2T>C; r.7826_8331del506; p.Gly2609Aspfs*4	1
			c.8400_8402delinsAAAA; p.Phe2801Lysfs*11	2
			c.8470A>T; p.Arg2824*	1
			c.8488-9T>G; r.8487_8488insTATTACAG; p.Trp2830Tyrfs*36	1
			c.8504C>A; p.Ser2835*	1
			c.8579delA; p.Lys2860Argfs*3	1
			c.8632+1656_8754+207del; r.8633_8754del122; p.Glu2878Glyfs*5	1
			c.8855dupT; p.Met2952Ilefs*5	1
			c.8961_8964delGAGT; p.Ser2988Phefs*12	1
			c.8969G>A; p.Trp2990*	1
			c.9070_9073delAACA; p.Asn3024Tyrfs*3	1
			c.9097delA; p.Thr3033Leufs*29	1
			c.9097dupA; p.Thr3033Asnfs*11	2

			c.9294C>G; p.Tyr3098*	7
			c.9354_9816del; r.9354_9816del463; p.Met3118Ilefs*3	1
			c.9382C>T; p.Arg3128*	2
			c.9393delC; p.Lys3132Asnfs*31	1
			c.9401delG; p.Gly3134Alafs*29	1
			c.9409dupA; p.Thr3137Asnfs*13	1
			c.10150C>T; p.Arg3384*	1
		CHEK2	c.247delC ; p.Gln83Lysfs*27	1
			c.909-391_1008+356del; p.Leu303Phefs*13	1
			c.917G>C ; p.Gly306Ala	1
			c.1072C>T; p.Gln358*	1
			c.1315C>T; p.Gln439*	1
			c.1461+1G>A; r.1376_1461del86; p.Ala459Glyfs*2	1
			deletion of exons 5-15	1
		PALB2	c.7G>T; p.Glu3*	1
			c.15delC; p.Lys7Serfs*11	1
			c.181C>T; p.Gln61*	1
			c.211+1G>A	3
			c.212-712_2587-888del9762; p.Pro72Serfs*20	1
			c.444delG; p.Lys149Serfs*28	1
			c.839delA; p.Asn280Thrfs*8	1
			c.857delC; p.Pro286Leufs*2	1
			c.1038delA; p.Glu347Asnfs*9	1
			c.1048C>T; p.Gln350*	1
			c.1059delA; p.Lys353Asnfs*3	6
			c.1240C>T; p.Arg414*	1
			c.1446delC; p.Ser483Hisfs*2	1
			c.1592delT; p.Leu531Cysfs*30	2
			c.1914dupT; p.Glu639*	1
			c.2016dupA; p.Glu673Argfs*42	1
			c.2108T>G; p.Leu703*	6
			c.2219_2220delAA; p.Gln740Argfs*4	1
			c.2594C>G; p.Ser865*	1
			c.2760dupA; p.Gln921Thrfs*7	2
			c.2968G>T; p.Glu990*	5

			c.3114-1G>A; r.3114_3350del237; p.Asn1039_Arg1117del79	1
			c.3114-2A>G; r.3114_3350del; p.Asn1039_Arg1117del	1
			r.3114_3350del237; p.Asn1039_Arg1117del79	1
			c.3201+2T>C; r.3114_3350del237; p.Asn1039_Arg1117del79	1
			c.3256C>T; p.Arg1086*	1
			c.3350+5G>A; r.3114_3350del237; p.Asn1039_Arg1117del79	1
			c.3507_3508delTC; p.His1170Phefs*19	1
		PTEN	c.1A>G; p.Met1Val	1
			c.388C>T; p.Arg130*	1
			c.407G>T; p.Cys136Phe	1
			c.491delA; p.Lys164Argfs*3	1
		TP53	c.96+1G>T; r.75_96del; p.Leu26Profs*11	1
			c.422G>A; p.Cys141Tyr	1
			c.473G>A; p.Arg158His	1
			c.490A>G; p.Lys164Glu	1
			c.524_547dup; p.Cys182_Ser183insCysCysProHisHisGluArgCys	1
			c.527G>T; p.Cys176Phe	1
			c.529_546del; p.Pro177_Cys182del	1
			c.536A>G; p.His179Arg	1
			c.541C>T; p.Arg181Cys	1
			c.626_627dupGA; p.Asn210Glufs*38	1
			c.722C>T; p.Ser241Phe	1
			c.743G>A; p.Arg248Gln	1
			c.775G>A; p.Asp259Asn	1
			c.818G>A; p.Arg273His	1
			c.825T>G; p.Cys275Trp	1
			c.844C>T; p.Arg282Trp	2
			c.848G>A; p.Arg283His	2
			c.854A>G; p.Glu285Gly	1
			c.916C>T; p.Arg306*	1
			c.1010G>A; p.Arg337His	1
			c.1025G>C; p.Arg342Pro	1

	Moderate & low	*APC*	c.7798_7801delCAAA; p.Gln2600Valfs*15	1
		ATM	c.1262C>G ; p.Ser421*	1
			c.2413C>T; p.Arg805*	1
			c.3397G>T; p.Glu1133*	1
			c.4668T>A ; p.Tyr1556*	1
			c.5170G>T ; p.Glu1724*	1
			c.5354del; p.Pro1785Leufs*8	1
			c.6115G>A; p.Glu2039Lys	1
			c.6910delG ; p.Glu2304Serfs*6	1
			c.7515+1G>T; r.7308_7515del208; p.Tyr2437Glufs*4	1
			c.8369del; p.Arg2790Asnfs*16	1
			c.8435_8436delCT; p.Ser2812Phefs*2	2
			c.8987+3G>A; r.?; p.?	1
		BAP1	c.718A>T; p.Lys240*	1
		BARD1	c.539_540delAT; p.Tyr180*	1
			c.1338C>A; p.Tyr446*	5
			c.623dupA; p.Lys209Glufs*5	1
			c.2167_2174delCATGCGAG; p.His723Thrfs*4	1
			del of whole gene (exons 1-11)	1
		BRIP1	c.1343G>A; p.Trp448*	2
			c.2392C>T; p.Arg798*	2
		MSH2	c.1457_1460delATGA; p.Asn486Thrfs*10	3
		MSH6	c.3013C>T; p.Arg1005*	1
		MUTYH	c.467G>A; p.Trp156*	4
			c.934-2A>G	1
		PMS2	c.1A>G ; p.Met1?	1
		RAD51C	c.1024A>T; p.Lys342*	1
			c.394dupA; p.Thr132Asnfs*23	4
		RAD51D	c.270_271dupTA; p.Lys91Ilefs*13	8
			c.801delC; p.Trp268Glyfs*42	1
Unselected Breast Cancer Control Patient (Fulfilled	High^	*BRCA1*	c.2599C>T; p.Gln867*	1
			c.3286C>T; p.Gln1096*	1
		BRCA2	c.774_775del; p.Glu260Serfs*15	1
			c.755_758del; p.Asp252Valfs*24	1
			c.7878G>A; p.Trp2626T*	1
		TP53	c.91G>A; p.Val31Ile	1

NCCN criteria)	Moderate & low	*ATM*	c.8817_8827del; p.Gln2942Valfs*10	1
		BARD1	c.448C>T; p.Arg150*	1
			c.1338C>A; p.Tyr446*	1
		BRIP1	c.1970del; p.Gly657Valfs*31	1
Unselected Breast Cancer Control Patient (Not fulfilled NCCN criteria)	High^	*BRCA1*	c.66_67insA; p.Glu23Argfs*18	1
			c.68A>G; p.Glu23Gly	1
			c.3286C>T; p.Gln1096*	1
			c.3858_3861del; p.Ser1286Argfs*20	1
		BRCA2	c.7878G>A; p.Trp2626*	1
	Moderate & low	*ATM*	c.4235C>T; p.Pro1412Leu	1
			c.5697C>A; p.Cys1899*	1
		BARD1	c.539_540del; p.Tyr180*	1
			c.1338C>A; p.Tyr446*	1
		MLH1	c.1756G>A; p.Ala586Thr	1
		RAD51D	c.271_272insTA; p.Lys91Ilefs*13	1
Normal Patient (with benign breast disease)	Moderate & low	*ATM*	c.205C>T; p.Gln69*	1
			c.1402_1403delAA; p.Lys468Glufs*18	1
			Del of exon 62-63	1
		BRIP1	c.1510dup; p.Ile504Asnfs*7	1
			c.1629-2A>G	1
		RAD51C	c.394dupA; p.Thr132Asnfs*23	1
		RAD51D	c.556C>T; p.Arg186*	1
		STK11	Del of exon 1-10	1

^High penetrance: *BRCA1, BRCA2, CHEK2, PALB2, TP53* and *PTEN*
